# Sol–Gel-Processed Y_2_O_3_–Al_2_O_3_ Mixed Oxide-Based Resistive Random-Access-Memory Devices

**DOI:** 10.3390/nano13172462

**Published:** 2023-08-31

**Authors:** Hae-In Kim, Taehun Lee, Yoonjin Cho, Sangwoo Lee, Won-Yong Lee, Kwangeun Kim, Jaewon Jang

**Affiliations:** 1 School of Electronic and Electrical Engineering, Kyungpook National University, Daegu 41566, Republic of Korea; khi5735@knu.ac.kr (H.-I.K.); lth0313@knu.ac.kr (T.L.); chongo815wls@knu.ac.kr (Y.C.); sangw98@knu.ac.kr (S.L.); yongsz@knu.ac.kr (W.-Y.L.); 2 The Institute of Electronic Technology, Kyungpook National University, Daegu 41566, Republic of Korea; 3 School of Electronics and Information Engineering, Korea Aerospace University, Goyang 10540, Republic of Korea

**Keywords:** Y_2_O_3_, Al_2_O_3_, sol–gel, resistive random-access-memory, high-resistance state, low-resistance state

## Abstract

Herein, sol–gel-processed Y_2_O_3_–Al_2_O_3_ mixed oxide-based resistive random-access-memory (RRAM) devices with different proportions of the involved Y_2_O_3_ and Al_2_O_3_ precursors were fabricated on indium tin oxide/glass substrates. The corresponding structural, chemical, and electrical properties were investigated. The fabricated devices exhibited conventional bipolar RRAM characteristics without requiring a high-voltage forming process. With an increase in the percentage of Al_2_O_3_ precursor above 50 mol%, the crystallinity reduced, with the amorphous phase increasing owing to internal stress. Moreover, with increasing Al_2_O_3_ percentage, the lattice oxygen percentage increased and the oxygen vacancy percentage decreased. A 50% Y_2_O_3_–50% Al_2_O_3_ mixed oxide-based RRAM device exhibited the maximum high-resistance-state/low-resistance-state (HRS/LRS) ratio, as required for a large readout margin and array size. Additionally, this device demonstrated good endurance characteristics, maintaining stability for approximately 100 cycles with a high HRS/LRS ratio (>10^4^). The HRS and LRS resistances were also retained up to 10^4^ s without considerable degradation.

## 1. Introduction

Properties such as high density, high speed, and cheap nonvolatile memory, as well as low-power operation, are the most crucial requirements for next-generation nonvolatile-memory technologies to replace conventional memory technology [1]. Many candidates are available with regard to this, such as resistive random-access memory (RRAM). RRAM is promising owing to its high switching speeds, simple device structure with high scalability, and multilevel storage properties, making it capable of being adapted to three-dimensional memory architectures, and has good compatibility with the conventional complementary metal–oxide–semiconductor (CMOS) fabrication process [2,3,4]. Recently, RRAM has shown the potential to realize neuromorphic systems, mimicking biological synapses similar to the human brain [5,6,7].

To realize RRAM devices, many metal oxides (such as ZrO_2_, HfO_2_, TiO_2_, and Y_2_O_3_) have been studied for use in the active channel layers of RRAM devices [8,9,10,11,12,13,14,15,16,17,18]. Y_2_O_3_ has been investigated to replace the low-k SiO_2_ dielectric layers with high-k ones during CMOS processes in industries. Moreover, Y_2_O_3_ has shown promising results in combining high-electron-mobility GaN transistors and SiC technology [19,20,21]. Notably, the fast ion movement inside RRAM Y_2_O_3_ layers is expected to lead to fast RRAM operation. However, the involved sneak-path problem remains a major concern to realize RRAM arrays. With regard to this, an additional transistor is connected to a single-RRAM device. Y_2_O_3_ passivation layers are employed for improving the bias stability of transistors. These layers can be simultaneously used as the active channel layers of Y_2_O_3_ RRAM devices, reducing the process steps and the fabrication cost for arrays with unit cells comprising one transistor and one RRAM [22,23]. As a base element, Y_2_O_3_ can be combined with Al_2_O_3_ to form various composites, such as Y_3_Al_5_O_12_, YAlO_3_, and Y_4_Al_2_O_9_. These Y_2_O_3_–Al_2_O_3_ composites are used as solid-state laser materials or reinforcement fibers in structural ceramics and intermetallic composites [24,25].

Several parameters, such as SET voltage, RESET voltage, low-resistance state (LRS), high-resistance state (HRS), endurance, and retention, are crucial for the performance evaluation of RRAM devices. Especially, the HRS/LRS ratio is a critical parameter to decide the readout margin and array size. A high HRS/LRS ratio implies a large readout margin and array size [26]. High HRS/LRS ratios are needed to realize the operation of multilevel cell (MLC) switching in RRAM [27,28]. Such high HRS/LRS ratios lead to an increased number of intermediate levels and improves the stability of each level for MLC switching operation, leading to a high bit density. The resistance between the top and bottom electrodes decides the LRS and HRS. The LRS is determined by the conductivity of the conductive path formed between these electrodes. Meanwhile, the HRS is dependent on the value of the leakage current inside the active materials between the electrodes. The leakage current is affected by the involved crystalline phase, defects inside the layers, or energy barrier heights [29,30,31]. Suppression of the HRS leakage current is critical for increasing the HRS/LRS ratio.

Herein, a sol–gel-processed Y_2_O_3_–Al_2_O_3_ mixed oxide was used for the active channel layer of RRAM devices. Various proportions of the Y_2_O_3_ and Al_2_O_3_ precursors involved were chosen. The resultant mixtures were investigated for their structural, chemical, and electrical properties, as well as memory characteristics, for the active channel layer. A 50% Y_2_O_3_–50% Al_2_O_3_ mixed oxide-based RRAM device exhibited the highest HRS/LRS ratio. Unlike pure Y_2_O_3_, Y_2_O_3_–Al_2_O_3_ composites suppressed the leakage current, enabling the determination of the HRS values, as well as leading to an improved HRS/LRS ratio, which was due to a transition of the involved polycrystalline films into an amorphous phase and reduced oxygen vacancies.

## 2. Materials and Methods

Thin films of Y_2_O_3_–Al_2_O_3_ mixed oxide were prepared on indium tin oxide (ITO)/glass substrates using the sol–gel spin-coating method. The Y_2_O_3_ precursor (0.6 M) was prepared by dissolving 1.04 g of yttrium (III) nitrate tetrahydrate (Y(NO_3_)_3_·4H_2_O; Sigma Aldrich, St. Louis, MO, USA, 99.9%) in 5 mL of 2-methoxyethanol (Sigma Aldrich, 99.8%). Meanwhile, the Al_2_O_3_ precursor involved dissolving 0.72 g of aluminum chloride hexahydrate (AlCl_3_·6H_2_O; Reagent Plus, 99%) in 5 mL of 2-methoxyethanol. These precursors were stirred at 80 °C for 1 h to obtain clear and homogeneous solutions. Several proportions of these precursors were used to obtain various solutions of Y_2_O_3_–Al_2_O_3_ mixed oxides (YAl-x, with x being 0%, 25%, 50%, 75%, and 100% molar ratios of Al_2_O_3_), and the resultant structural, chemical, and RRAM characteristics were investigated. Prior to the application of the coating method, the ITO/glass substrates were washed with acetone and deionized water (DI) (each for 10 min) via sonication. Further, an ultraviolet/ozone treatment was conducted for 1 h to eliminate organic contaminants, such as particles, residues, and volatile organic compounds, from the substrates. Then, the prepared solutions were uniformly coated on the clean substrates using the sol–gel spin-coating method at 3000 rpm for 50 s. The coated thin films were baked on a hot plate at 150 °C for 10 min and then annealed in a furnace at 500 °C for 2 h. Subsequently, a thermal evaporator was used to deposit 100 nm Ag top electrodes on the coated films. The deposition was performed using a patterning mask at a pressure of 5.0 × 10^−6^ Torr and deposition rate of 1.8 Å/s to form 30 μm × 30 μm Ag electrodes.

The crystal structures and crystallographic orientations of the films were investigated using grazing-incidence X-ray diffraction (GIXRD, X’pert Pro, Malvern PANalytical, Malvern, UK) with Cu kα radiation (*λ* = 1.54 Å). Field-emission scanning electron microscopy (FESEM, S-4800, Hitachi, Cold type, Tokyo, Japan) was used to estimate the film thickness and surface roughness. The elemental composition and chemical states of the films were analyzed using X-ray photoelectron spectroscopy (XPS; NEXSA, Thermo Fisher, Waltham, MA, USA) with monochromatic Al Kα (1486.6 eV). A probe station (MST T-40000A, Hwaseong, Republic of Korea) with a source measurement unit (KEITHLEY 2636B) was employed to assess the electrical properties of the fabricated RRAM devices.

## 3. Results and Discussion

**Figure 1:**
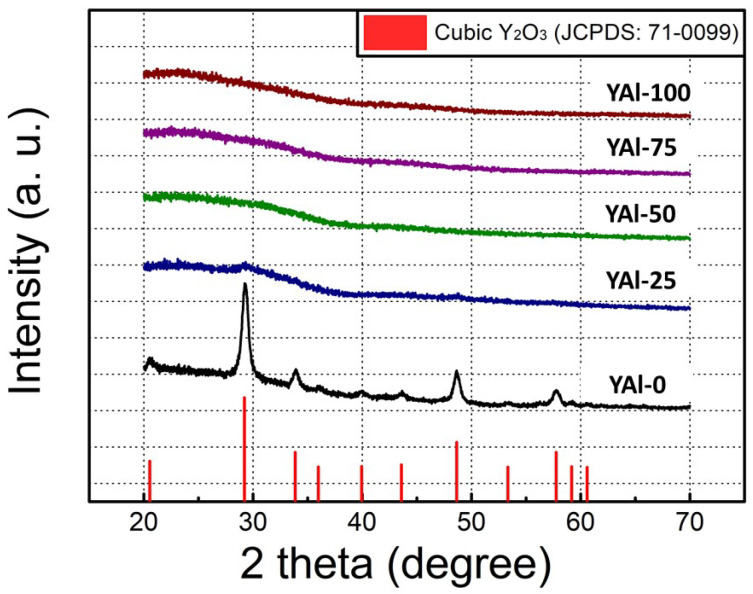
GIXRD spectra of sol–gel-processed Y_2_O_3_–Al_2_O_3_ mixed oxide films with varying Al_2_O_3_ content.

Figure 1 presents the GIXRD patterns of the prepared films. GIXRD analysis was used to investigate the changes in the film crystalline structure with the molar ratio. The GIXRD patterns revealed that the YAl-0 film preferentially exhibits a (222)-oriented polycrystalline phase. The diffraction peaks observed at 2*θ* values of 29.30°, 33.96°, 48.79°, and 57.93° correspond to the (222), (400), (440), and (622) planes, respectively, of the Y_2_O_3_ cubic structure (JCPDS 74-1828). Metastable monoclinic Y_2_O_3_ can be formed at low temperatures [32]. However, herein, a low-temperature-stability cubic Y_2_O_3_ phase is dominant. The presence of the main peak at 29.30° indicates that the grains primarily grow in the (222) plane. Meanwhile, the GIXRD pattern of the YAl-25 film showed weakened diffraction peaks of Y_2_O_3_ because of the defects caused by the difference in the radii of Al^3+^ (57 pm) and Y^3+^ (104 pm). A higher proportion of Al_2_O_3_ leads to a smaller crystalline size of Y_2_O_3_ compared to that corresponding to the YAl-0 film. The other three cases showed an amorphous phase. The crystalline sizes of each film can be calculated using the Scherrer equation:

*D* = (0.9 *λ*)/(*β* Cos *θ*), (1)

where *D* is the crystalline size, *λ* is the X-ray wavelength, *β* is the full-width half maximum value, and *θ* is the Bragg diffraction angle. The average crystalline size corresponding to the (222) plane was calculated to be 12.49 nm for the YAl-0 film and 4.24 nm for the YAl-25 film.

**Figure 2:**
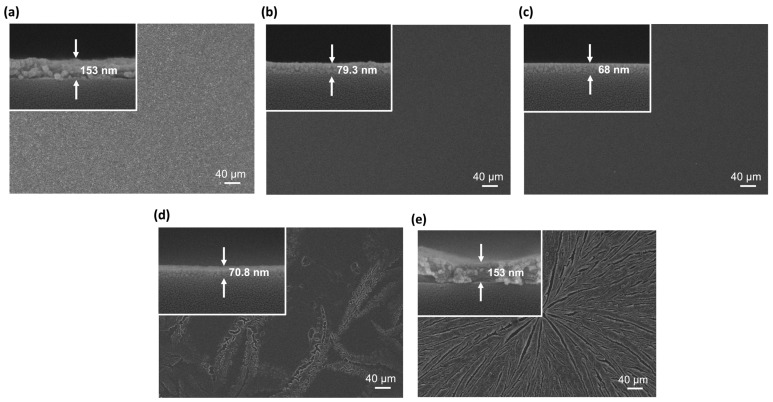
FESEM images of the film surface and cross-section (in the inset): (**a**) YAl-0, (**b**) YAl-25, (**c**) YAl-50, (**d**) YAl-75, and (**e**) YAl-100.

The film thickness and surface morphology are presented in Figure 2. The final thicknesses of the YAl-0, -25, -50, -75, and -100 films were 153, 79.3, 68, 70.8, and 153 nm, respectively. The Al_2_O_3_-rich films—YAl-75 and YAl-100—exhibited some pinholes and cracks.

**Figure 3:**
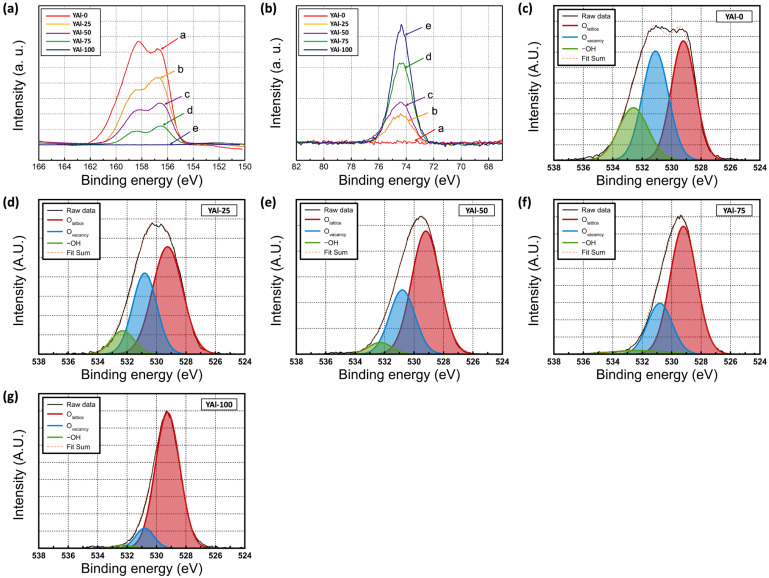
XPS spectra for YAl-X samples in the high-resolution windows for (**a**) Y 3d, (**b**) Al 2p, and (**c**–**g**) O 1s of sol–gel-processed Y_2_O_3_–Al_2_O_3_ mixed oxide films as functions of the Al_2_O_3_ content.

Figure 3 shown the high-resolution XPS spectra of the films. The results of the corresponding analysis are presented in Table 1. Figure 3a presents the high-resolution XPS spectra for Y 3d, revealing two splitting orbitals—Y 3d_5/2_ and Y 3d_3/2_. The corresponding peaks are located at 156.5 and 158.5 eV, respectively, indicating the formation of Y_2_O_3_. Figure 3b presents the high-resolution XPS spectra for Al 2p, indicating the formation of Al_2_O_3_. The atomic percentage of Al 2p increased with increasing Al_2_O_3_ content. The deconvolutions performed for analysis of O 1s are depicted in Figure 3c–g. The fitting results presented in the figures reveal three peaks at 529.2, 530.8, and 532.3 eV, which correspond to lattice oxygen, oxygen vacancies, and hydroxyl groups, respectively. With increasing Al_2_O_3_ content, the O_L_ percentage increased and the O_V_ percentage decreased.

**Figure 4:**
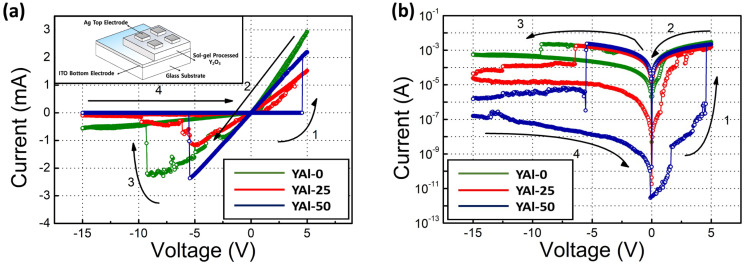
Representative I–V characteristics of sol–gel-processed Y_2_O_3_–Al_2_O_3_ mixed oxide-based RRAM devices as functions of the Al_2_O_3_ content: (**a**) linear scale, the inset showed the schematic image of the fabricated RRAM devices, and (**b**) log scale. The arrows and numbers indicate the voltage sweep directions.

Figure 4 presents the electrical characteristics of the fabricated devices. The YAl-0, -25, and -50 film-based devices exhibited the conventional bipolar resistive switching behavior. However, the devices fabricated using the YAl-75 and -100 films exhibited only the properties of linear and short I–V (not shown here) and no conventional properties of RRAM devices. The FESEM images of the YAl-75 and pure Al_2_O_3_ films revealed considerable clear cracks, which led to a short IV, indicating a direct connection between the top and bottom electrodes through pinholes or cracks. The YAl-0, -25, and -50 film-based RRAM devices did not undergo a forming process. Notably, metal-filament-based RRAM devices with Ag or Cu as top electrodes or RRAM devices with oxygen vacancy-rich materials do not require an initial forming process [33,34].

Figure 4a,b depict the resistive switching behavior of the prepared RRAM devices. On the application of appropriate voltage pulses, the devices can alternate between HRS and LRS, thereby enabling data storage. The prepared RRAM devices start from their HRS. When a specific positive voltage is applied, the current rapidly increases, indicating the formation of conductive filaments. This voltage is referred to as SET voltage; it converts the device state into LRS. Conversely, when a specific negative voltage is applied, the current abruptly decreases, indicating the removal of conductive filaments. This voltage is referred to as RESET voltage; it returns the device state into HRS. Depending on the composition of the conductive filaments, RRAM can be classified into conductive-bridge random-access memory (CBRAM) and oxygen-vacancy-based RRAM (OxRRAM). While CBRAM relies on the migration of metal ions, OxRRAM depends on the formation of oxygen vacancies and subsequent migration of oxygen ions. In a previous study, the authors confirmed that RRAM devices with a Ag/Y_2_O_3_/ITO structure belong to a type of CBRAM [14]. During the programming operation, a positive voltage bias is applied to the Ag top electrode. This bias induces the formation of a conductive filament within the Y_2_O_3_ layer. The Ag electrode acts as a source of metal ions that are electrochemically driven into the Y_2_O_3_ layer owing to the applied voltage. After migrating to the Y_2_O_3_ layer, these ions form conductive filaments between the Ag and ITO electrodes. These filaments represent an LRS and enable high current conduction. In contrast, during the erase operation, a reverse voltage is applied, causing the ions to migrate back to the Ag electrode, effectively breaking the existing conductive filaments. This results in an HRS, and the flow of current is restricted.

**Figure 5:**
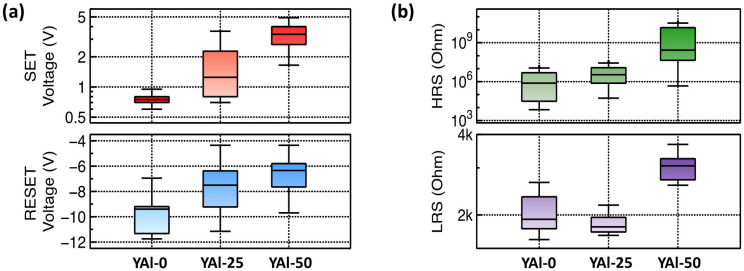
(**a**) SET (Red)/RESET (Blues) voltages (linear scale) and (**b**) HRS (Green) and LRS (Purple) resistances (log scale) of sol–gel-processed Y_2_O_3_–Al_2_O_3_ mixed oxide-based RRAM devices.

Figure 5a depicts the SET and RESET voltages of the fabricated RRAM devices as functions of the Al_2_O_3_ content. With increasing Al_2_O_3_ content, the SET voltage increased, while the absolute value of the RESET voltage decreased. With regard to the film crystal structure, as the grain size decreased and the number of grain boundaries increased, the SET voltage decreased. Notably, this was because of the ease in the formation of conductive filaments along grain boundaries [35,36]. The YAl-0 film-based RRAM device with the largest grain size in the insulating layer exhibited the lowest SET voltage, indicating that the concentration of oxygen vacancies is the most critical factor influencing the SET voltage. Oxygen vacancy sites can also serve as pathways for filament formation, as Ag ions can migrate through these sites with low migration barriers, requiring less energy for their movement [14,37]. Therefore, in the YAl-50 film-based device with the lowest concentration of oxygen vacancies, the migration of Ag ions was restricted, resulting in the highest SET voltage. Furthermore, the limited migration of Ag ions reduced the number of Ag conductive filaments. Consequently, less energy was required to eliminate the filaments, resulting in a low RESET voltage. In Figure 5b, the HRS and LRS resistances of the fabricated RRAM devices are depicted as functions of the Al_2_O_3_ content. The LRS resistances of all the devices remained relatively unchanged at ~10^3^ Ω, whereas the HRS resistances varied from 5.4 × 10^5^ (YAl-0) to 1.1 × 10^9^ Ω (YAl-50). Under the HRS condition, the film quality between the top and bottom electrodes determines the magnitude of the leakage current corresponding to the HRS resistance. Oxygen vacancies contribute to an increase in the leakage current because they introduce deep-trap energy levels, enabling the activation of mobile electrons. Furthermore, increasing grain sizes lead to an increase in the leakage current and a reduction in the number of grain boundaries, which serve as scattering sites for charge carriers. This results in an increase in the leakage current [29]. Therefore, the YAl-0 film-based device, with the highest oxygen vacancy concentration and the largest grain size, exhibited the lowest HRS resistance. Table 2 presents a comparison between the performances of the prepared RRAM devices and RRAM devices with only Y_2_O_3_. As can be seen, the HRS/LRS ratios of the prepared RRAM devices were considerably higher. Still, there are some uniformity issues. These originate from the randomly formed multiple conductive paths. 

To address these issues, smaller electrodes would be helpful to suppress the number of random formations and the growth of the conductive path. In addition, hourglass-shaped electrodes or surface-roughness-enhanced active channel layers were used to enhance local electrical field and improve device reliability [15,41,42,43].

**Figure 6:**
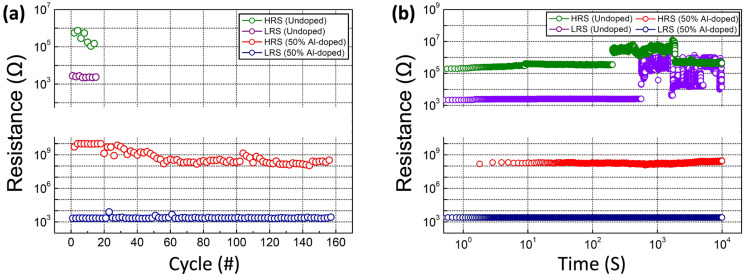
Nonvolatile-memory properties of the YAl-0 and -50 film-based devices: (**a**) endurance and (**b**) retention.

The endurance and retention characteristics of the fabricated devices were assessed to evaluate their nonvolatile-memory properties. After the programming and erase operations (each lasting for 50 ms), the LRS and HRS resistances were measured at a voltage of +0.1 V. As shown in Figure 6a), the YAl-0 film-based RRAM device exhibited poor endurance characteristics (<20 cycles) with a low HRS/LRS ratio. Excessive oxygen vacancies might have contributed to the excessive formation of Ag conductive filaments, leading to the failure of the RESET process [14]. In contrast, the YAl-50 film-based RRAM device exhibited good endurance characteristics, maintaining stability for approximately 100 cycles, with a high HRS/LRS ratio (>10^5^). Furthermore, with regard to retention characteristics, the HRS and LRS resistances of the YAl-50 film-based device remained fairly uniform up to 10^4^ s without substantial degradation, while those of the YAl-0 film-based device exhibited considerable degradation in HRS and LRS resistances before reaching 10^3^ s (Figure 6b).

## 4. Conclusions

Herein, sol–gel-processed Y_2_O_3_–Al_2_O_3_ mixed oxide-based RRAM devices were fabricated on ITO/glass substrates. With an increase in the content of Al_2_O_3_, the crystallinity reduced. An amorphous phase was present in the films with an Al_2_O_3_ content of 50% and above due to internal stress. This phase resulted from the difference between the ionic radii of Y and Al. With increasing Al_2_O_3_ content, the O_L_ percentage increased and the O_V_ percentage decreased. The presence of an amorphous phase and a low O_V_ concentration successfully decreased the leakage current under the HRS condition, leading to high HRS/LRS ratios, as required for a large readout margin and array size. Among the prepared devices, the YAl-50 film-based RRAM device exhibited the best endurance characteristics, maintaining stability for approximately 100 cycles with a high HRS/LRS ratio (>10^5^), and retention characteristics, exhibiting uniform HRS and LRS resistances up to 10^4^ s without considerable degradation.

## Figures and Tables

**Table 1:** XPS analysis of the prepared films.

Sample	at%			Y 3d		O 1s			Y/(Y + Al)	
	Al 2p	O 1s	Y 3d	Y_2_O_3_%	Y-CO%	O_L_	O_V_	–OH	exp	nom
YAl-0	-	66.3	31.3	63.2	36.8	39.9	38.9	21.2	1.0	1.0
YAl-25	11.5	64.5	24.1	71.2	28.8	56.3	34.3	9.4	0.68	0.75
YAl-50	17.1	65.3	17.6	83.3	16.7	64.6	30.2	5.1	0.51	0.5
YAl-75	27.5	63.9	8.6	96.8	3.2	70.1	25.9	4.0	0.24	0.25
YAl-100	36.6	63.2	-	-	-	88.7	9.8	1.5	0.0	0.0

**Table 2:** Performances of Y_2_O_3_-based and the prepared RRAM devices.

References	Structure	V_SET_ (V)	V_RESET_ (V)	HRS/LRS	Endurance (Cycle)	Retention (s)
[18]	ITO/Y_2_O_3_/Ag	+1.5 V	−15.0 V	~10^4^	~10^2^	~10^3^
[38]	TiN/Y_2_O_3_/Pt	−1.0 V	+1.0 V	~10^2^	∼8 × 10^2^	~10^5^
[39]	Al/Y_2_O_3_/Al	+1.74 V	−0.8 V	~30	~3 × 10^4^	~10^5^
[40]	n-Si/a-Y_2_O_3_/Y_2_O_3_/Al	+6.0 V	−6.0 V	~10	~3 × 10^4^	~10^3^
This work	ITO/Y_2_O_3_–Al_2_O_3_/Ag	+3.5 V	−6.5 V	~10^5^	~10^2^	~10^3^

## Data Availability

Data are available in a publicly accessible repository.
